# LPS-Induced Modifications in Macrophage Transcript and Secretion Profiles Are Linked to Muscle Wasting and Glucose Intolerance

**DOI:** 10.4014/jmb.2309.09037

**Published:** 2023-12-01

**Authors:** Heeyeon Ryu, Hyeon Hak Jeong, Seungjun Lee, Min-Kyeong Lee, Myeong-Jin Kim, Bonggi Lee

**Affiliations:** 1 Department of Food Science and Nutrition, Pukyong National University, Busan 48513, Republic of Korea; 2 Department of Smart Green Technology Engineering, Pukyong National University, Busan 48513, Republic of Korea

**Keywords:** Macrophages, skeletal muscle, cytokine, RNA Seq, proteome array

## Abstract

Macrophages are versatile immune cells that play crucial roles in tissue repair, immune defense, and the regulation of immune responses. In the context of skeletal muscle, they are vital for maintaining muscle homeostasis but macrophage-induced chronic inflammation can lead to muscle dysfunction, resulting in skeletal muscle atrophy characterized by reduced muscle mass and impaired insulin regulation and glucose uptake. Although the involvement of macrophage-secreted factors in inflammation-induced muscle atrophy is well-established, the precise intracellular signaling pathways and secretion factors affecting skeletal muscle homeostasis require further investigation. This study aimed to explore the regulation of macrophage-secreted factors and their impact on muscle atrophy and glucose metabolism. By employing RNA sequencing (RNA-seq) and proteome array, we uncovered that factors secreted by lipopolysaccharide (LPS)-stimulated macrophages upregulated markers of muscle atrophy and pro-inflammatory cytokines, while concurrently reducing glucose uptake in muscle cells. The RNA-seq analysis identified alterations in gene expression patterns associated with immune system pathways and nutrient metabolism. The utilization of gene ontology (GO) analysis and proteome array with macrophage-conditioned media revealed the involvement of macrophage-secreted cytokines and chemokines associated with muscle atrophy. These findings offer valuable insights into the regulatory mechanisms of macrophage-secreted factors and their contributions to muscle-related diseases.

## Introduction

Macrophages are multifunctional immune cells that serve various purposes in the body, including tissue repair and regeneration, immune defense, and immune response regulation [1-3]. These cells are present in virtually all tissues, where they exhibit diverse shapes and functions [4, 5]. In skeletal muscle, macrophages play a vital role in maintaining tissue homeostasis and promoting growth and regeneration [6]. Their effects on muscle function vary depending on the context of inflammation [7, 8]. During acute inflammation, such as injury, macrophages help repair and remove damaged cells and debris, recruit immune cells, and stimulate muscle precursor cell growth and differentiation by producing cytokines and chemokines [9, 10]. However, chronic inflammation can lead to muscle wasting through protein breakdown caused by the release of pro-inflammatory cytokines, ultimately resulting in immune tissue damage and dysfunction [11].

Skeletal muscle atrophy, characterized by a reduction in muscle mass, can occur due to an imbalance between protein synthesis and breakdown, triggered by various factors, such as aging, disease, and chronic inflammation [12-14]. Muscle wasting can result in insulin resistance, impaired blood glucose control, and reduced glucose uptake due to the decrease in glucose transporters on the cell surface [15]. Therefore, individuals with muscle atrophy may require higher insulin levels to regulate their blood glucose levels [16]. Moreover, the underlying mechanism of these diseases involves abnormal immune responses and inflammation, which can trigger the secretion of various factors by macrophages. These factors can further exacerbate muscle damage and wasting, thereby contributing to the progression of the disease [17, 18]. Therefore, it is crucial to understand how macrophage-secreted factors are regulated to comprehend the mechanism of these diseases.

The inflammatory response of macrophages is commonly investigated by using lipopolysaccharide (LPS), a component of the outer membrane of gram-negative bacteria [19]. LPS activates macrophages, prompting them to produce various cytokines and chemokines [20]. However, the specific signaling pathways altered by LPS and the effects of numerous factors secreted by LPS-stimulated macrophages on tissues such as skeletal muscle are not well understood. Our study utilized RNA sequencing analysis (RNA-seq) and proteome array technologies to identify signaling pathways regulated by LPS-stimulated macrophages and to investigate their effects on skeletal muscle atrophy and glucose metabolism. We aim to identify macrophage-secreted factors that impact muscle atrophy and glucose metabolism and uncover novel causes of muscle-related diseases.

## Materials and Methods

### RAW 264.7 Macrophage Cell Culture and Preparation of Conditioned Medium

The RAW 264.7 macrophage cell line was obtained from the Korea Cell Line Bank and cultured in Dulbeccós modified Eaglés medium (DMEM) supplemented with 10% Fetal Bovine Serum (FBS) and 1% penicillin-streptomycin (P/S) under 5% CO2 humidified conditions at 37°C. Cells were seeded at a density of 4 × 10^5^ cells per well in 12-well culture plates and allowed to reach 80% confluency. After treatment with LPS (*E. coli* O111:B4) (or left untreated) for 3 h, the cells were washed twice with Dulbeccós phosphate-buffered saline (DPBS) and incubated in a serum-free medium for 24 h to generate macrophage-conditioned media (MCM). The MCM from both LPS-stimulated (LPS-MCM) and untreated (CON-MCM) cell media was collected, and centrifuged at 1,000 rpm for 3 min, and the supernatant was stored at -80°C for further experiments. For further experiments, 20% of MCM was treated in C2C12 cells.

### C2C12 Skeletal Muscle Cell Culture and Differentiation

The C2C12 skeletal muscle cells were acquired from the American Type Culture Collection (ATCC, USA) and cultured in DMEM medium supplemented with 10% FBS and 1% P/S under 5% CO2 humidified conditions at 37°C. The cells were seeded at a density of 2 × 10^5^ cells per well in a 6-well culture plate and allowed to reach 90%confluence. To induce differentiation into myotubes, the serum concentration was reduced from 10% FBS to 2%FBS. The medium was refreshed every 2 days, and the cells were used on the fifth day of differentiation.

### Myotube Diameter Measurement

The diameters of the C2C12 differentiated myotubes were determined using Giemsa staining. On the fifth day of cell differentiation, previously collected MCMs were added to the cells and incubated for 24 h. The cultured cells were then fixed with a 4% Paraformaldehyde Phosphate Buffer Solution (#161-20141, Fujifilm WAKO, USA) for 10 min, followed by staining with the Giemsa solution (Sigma-Aldrich, USA) for 45 min and thoroughly washing with distilled water. After air-drying, at least six random images were captured from each well. Using ImageJ software (version 1.53t; National Institutes of Health), the diameters of 50 to 100 myotubes were measured in each well.

### 2-Deoxy Glucose Uptake

The Glucose Uptake Assay Kit (ab136955, Abcam, UK) was utilized to measure glucose uptake following the manufacturer's protocol. Differentiated C2C12 cells were treated with MCMs for 24 h, then starved for 40 min using Krebs-Ringer-Phosphate-Hepes (KRPH) buffer (containing 20 mM HEPES, 5 mM KH2PO4, 1 mM MgSO4, 1 mM CaCl2, 136 mM NaCl, 4.7 mM KCl, pH 7.4). After the starvation period, the cells were stimulated with or without 100 nM insulin for 20 min, followed by incubation with 10 μl of 2-deoxy-glucose (2-DG) for 20 min. The oxidation reaction of 2-DG-6-phosphate (2-DG6P) to NADPH was measured at 412 nm using a microplate reader (AMR-100, All-sheng, China) at 37°C.

### Real Time-PCR

RNA was extracted from the cells using the RiboEXTM reagent (GeneAll, Republic of Korea) according to the manufacturer's instructions. The extracted RNA was converted to cDNA using the SmartGene compact cDNA Synthesis kit (SMART GENE, Republic of Korea). Real-time PCR was performed using the TOPrealTM SYBR Green qPCR PreMIX (Enzynomics, Republic of Korea) on a QuantStudio™ 1 Real-Time PCR system (Applied Biosystems, USA). The expression levels of the target genes were normalized to the housekeeping gene beta-actin, and the fold change was calculated using the 2^-ΔΔCT^ method [21]. The primer sequence will be provided upon request.

### RNA Purification and Sequencing

Total RNA was isolated from RAW 264.7 cells using the RiboEX™ reagent (GeneAll). RNA integrity was assessed using an Agilent 2200 TapeStation (Agilent Technologies, USA), and preparations with a RIN value of 7 or higher were used for RNA library construction. Libraries were purified and generated following the manufacturer's guided steps in the Illumina TruSeq Stranded Total RNA Library Prep Gold Kit (Illumina, USA, #20020599). Briefly, ribosomal RNA present in total RNA was removed using the Ribo-Zero Gold rRNA Removal Kit (Human/Mouse/Rat) (Illumina). After synthesizing first- and second-strand cDNAs from purified RNA samples, adapter ligation was performed to generate a cDNA library. The cDNA library was quantified using the KAPA Library Quantification Kit for the Illumina Sequencing platform according to the qPCR Quantification Protocol Guide (KAPA BIOSYSTEMS, #KK4854), and library quality was checked using the TapeStation D1000 ScreenTape (Agilent Technologies, # 5067-5582). Paired-end (2 × 151 bp) sequencing was performed using the Illumina NovaSeq6000 (Illumina, Inc.).

### RNA-Seq Analysis

Raw reads were preprocessed to remove low-quality and adapter sequences and mapped to Mus musculus (mm10) using HISAT (version 2.1.0) [22, 23]. The reference genome sequence and annotation data of Mus musculus (mm10) were obtained from NCBI. Then, transcript assembly was performed using StringTie (version 2.1.3b) [22, 24].

### Analysis of Differentially Expressed Genes

To identify differentially expressed genes, statistical analysis was performed using the edge R package (version 3.40) [25]. Briefly, genes with one or more zero read count values were excluded from the raw count data. Filtered data were log2 transformed and subjected to the TMM normalization method. The TMM normalization referred to the no replicates (square-root-dispersio*n* = 0.1) method of "edgeR: Differential analysis of sequence read count data User's Guide". Data statistical significance was determined using the Benjamini-Hochberg algorithm for False Discovery Rate (FDR) adjustments for p-values. Differentially expressed genes were screened using the condition |log2 Fold change| >=1, FDR < 0.05. Gene ontology (GO) and KEGG enrichment analysis were performed using clusterProfiler (version 4.7.1.003) in the R package [25].

### Cytokine and Chemokine Profile Analysis

Cytokine and chemokine levels were analyzed using the Proteome Profiler Mouse XL Cytokine Array Kit (R&D, USA) following the manufacturer's instructions. Briefly, the membranes were incubated with a blocking buffer for 1 h at room temperature. Next, the membrane was incubated with the biotinylated Detection Antibody Cocktail and MCMs overnight at a temperature ranging from 2-8°C. After washing, the membrane was incubated with Streptavidin-HRP for 30 min at room temperature. Finally, the membranes were exposed to Chemi Reagent Mix for 1 min and X-ray film for 10 min. The resulting images were analyzed using Quick Spots Tool software (R&D) for quantification. The expression levels of cytokines and chemokines were reported as signal intensities normalized to the reference spots on the same membrane.

### Statistical Analysis

The results of the experiments were expressed as the mean ± standard error of the mean (SEM) and were based on data collected from at least three independent trials. The significance between groups was analyzed using GraphPad Prism 5.0 software (GraphPad Software, USA), with one-way analysis of variance (ANOVA) and Tukey's multiple comparison test.

## Results

### Effects of LPS-Macrophage-Conditioned Medium (MCM) on Muscle Atrophy, Inflammatory Responses, and Glucose Uptake in C2C12 Myotubes

**Fig. 1:**
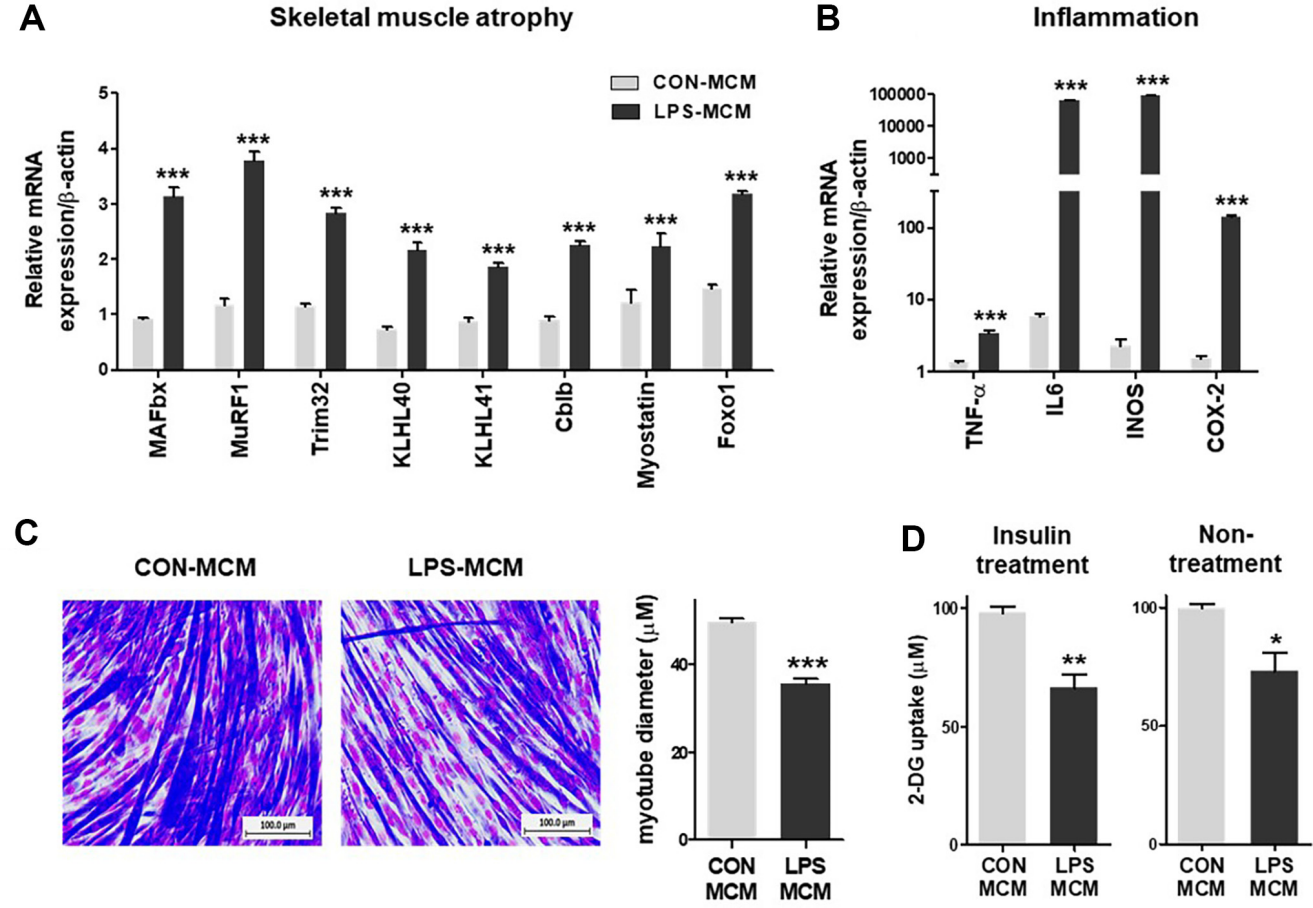
Effects of LPS-MCM on myotube diameter muscle atrophy, and inflammation expression in C2C12 myotubes. C2C12 myotube cells were treated with 20% MCM (LPS or control) for 24 h. (**A**) mRNA expression levels of E3 ubiquitin ligase and (**B**) pro-inflammatory cytokines and mediators were measured by real-time PCR. (**C**) Representative images of myotubes captured at ×20 magnification, showing differences in root canal diameters between CON-MCM and LPSMCM treatment groups. (**D**) Glucose absorption capacity levels were measured in both insulin-treated and untreated groups. Data are presented as mean ± SEM. Statistical analysis was performed using separate tests for each gene, and the significance levels were indicated as follows: *p < 0.05, **p < 0.01, ***p < 0.001 compared with the CON-MCM group.

To examine the impact of macrophage-derived secretion factors on muscle function, we conducted experiments using differentiated C2C12 myotubes exposed to LPS-treated macrophage-conditioned medium (LPS-MCM) and normal macrophage-conditioned medium (CON-MCM). Our focus was to assess changes in muscle atrophy and inflammatory responses induced by macrophage-derived secretion factors. Analysis of markers associated with the ubiquitin-proteasome pathway revealed a significant upregulation of several E3 ubiquitin ligases, including muscle RING-finger protein-1 (MuRF1), muscle atrophy F-box (MAFbx), tripartite motif-containing protein (Trim)-32, cbl Proto-oncogene b (Cblb), kelch-like family member (KLHL) 40, and KLHL41, in response to LPS-MCM treatment, thus activating the ubiquitin-proteasome pathway (Fig. 1A) [26, 27]. Furthermore, LPS-MCM treatment increased the mRNA expression of pro-inflammatory cytokines and mediators, such as tumor necrosis factor (TNF)-α, interleukin (IL)-6, inducible nitric oxide synthase (iNOS), and cyclooxygenase (COX)-2 (Fig. 1B), indicating the induction of an inflammatory response in muscle cells. Additionally, we observed a 27.9%reduction in cell diameter in LPS-MCM-treated myotubes compared to CON-MCM (Fig. 1C), suggesting the potential for muscle atrophy. To investigate the effect of MCM on glucose uptake in C2C12 myotubes, we measured 2-deoxy-D-glucose (2-DG) uptake. Our results revealed a significant reduction of 32.5% in insulin-stimulated conditions and 26.7% in non-insulin-stimulated conditions in the presence of LPS-MCM compared to CON-MCM (Fig. 1D), indicating a potential interference with glucose uptake and impact on glycemic control.

### LPS-Stimulated Macrophages Exhibit Extensive Alterations in Gene Expression Patterns

**Fig. 2:**
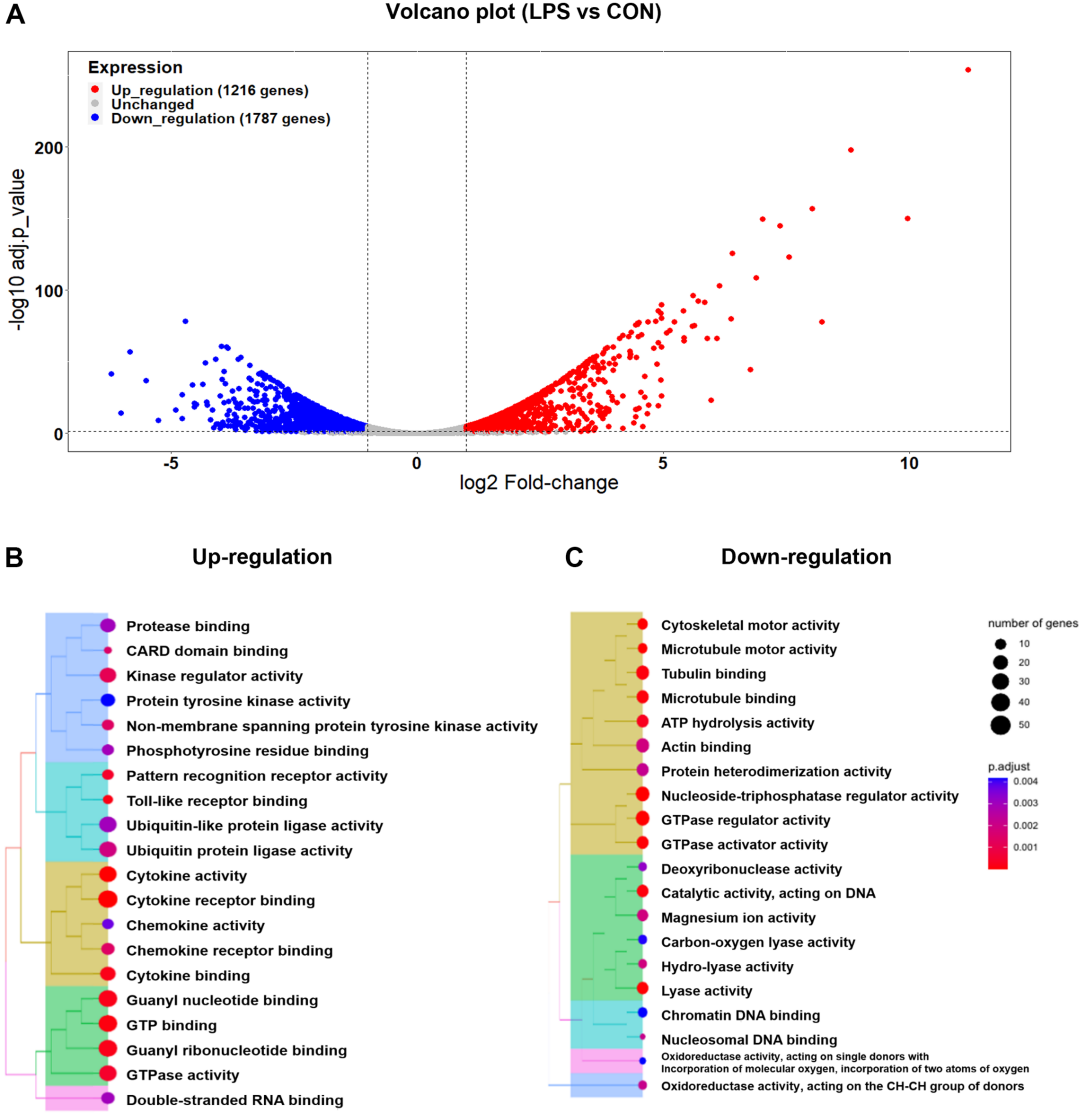
Analysis of differentially expressed genes and GO in LPS-stimulated macrophages. (**A**) The volcano plot revealed 1216 upregulated genes (red) and 1787 downregulated genes (blue). Analysis of GO was focused on molecular functions, and it identified (**B**) GO terms associated with upregulated genes and (**C**) GO terms associated with downregulated genes. Both the volcano plot and GO analysis were conducted using a threshold of |log2 fold change| >= 1 and FDR < 0.05.

Given the significant effects of MCM on muscle volume and glucose homeostasis-associated gene profile in C2C12 myotubes, we performed RNA-seq analysis to analyze the gene expression profiles of macrophages treated with LPS. The analysis revealed a total of 15,816 normalized genes, with 1,216 genes showing up-regulation and 1,787 genes showing down-regulation (Fig. 2A). Importantly, the differentially expressed genes extended beyond immune system-related genes and encompassed a wide array of signaling pathways associated with carbohydrate metabolism, fatty acid beta-oxidation, and other nutritional metabolic processes. Notably, genes involved in carbohydrate metabolism, such as acetyl-CoA synthetase, glutamine synthetase, and aldehyde dehydrogenase, showed altered expression levels. Key genes related to fatty acid beta-oxidation, including acetyl-Coenzyme A acyltransferase 2, acyl-Coenzyme A dehydrogenase, and carnitine palmitoyltransferase 1alpha and beta, also exhibited differential expression. Furthermore, several genes associated with insulin endocrine signalings, such as acetyl-Coenzyme A carboxylase beta, TBC1 domain family member 4, and ras-related protein Rab-3A, were affected (Fig. S1A). These findings suggest that LPS stimulation not only activates the immune system in macrophages but also potentially influences nutrient metabolism pathways. To gain a more comprehensive understanding of the specific changes in gene expression, we performed a KEGG pathway analysis, which identified enriched pathways among the upregulated genes (Fig. S2). Notably, we observed alterations in various immune system pathways, including the toll-like receptor signaling pathway. Moreover, pathways associated with the activity of cytokines and chemokines, known secreted factors, were abundantly represented among the differentially expressed genes (Fig. S2A and S2B).

### Analysis of GO and Network for Secreted Factors in LPS-Stimulated Macrophages

**Fig. 3:**
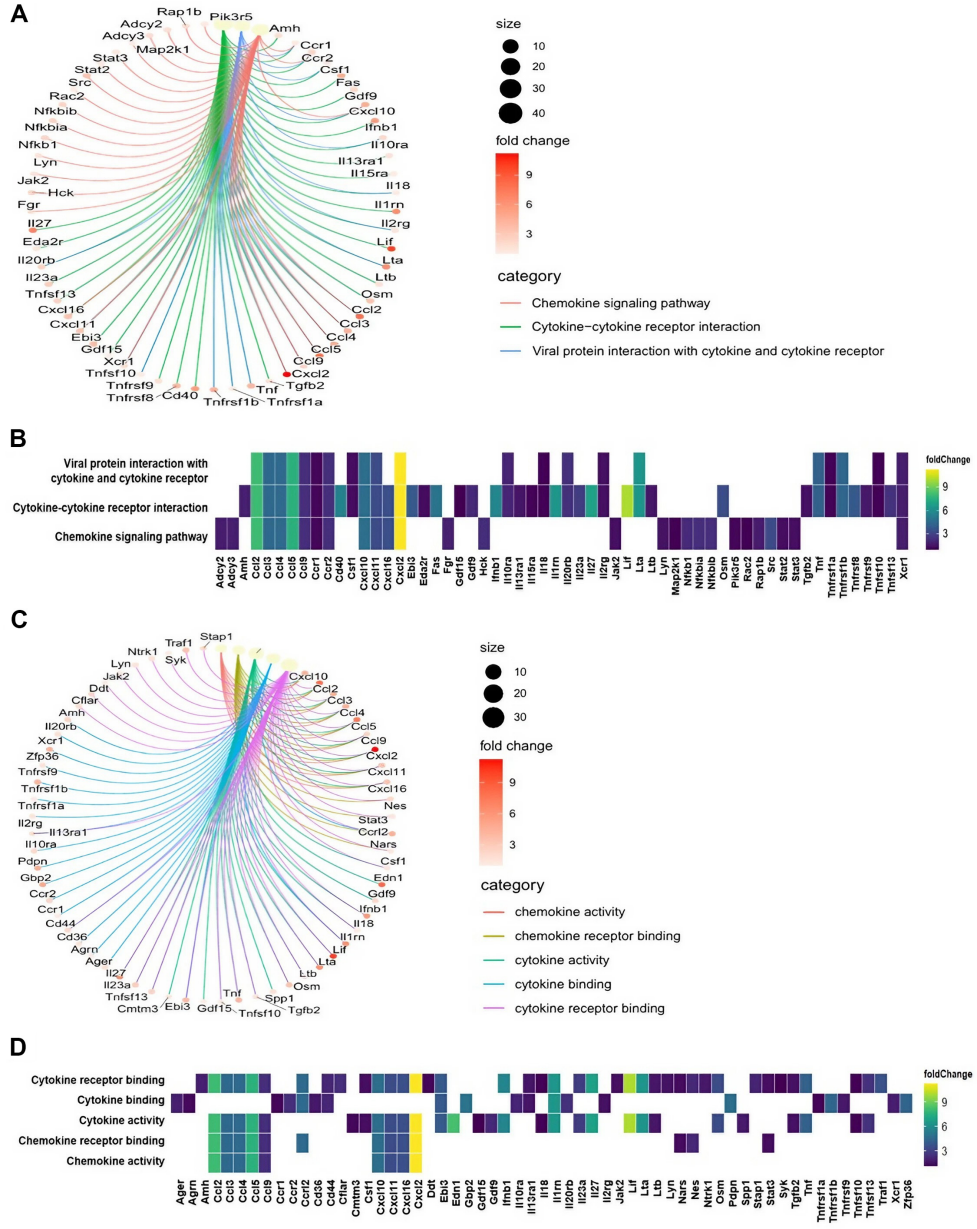
Network analysis of secreted factors associated with GO terms and KEGG pathway categories. A heatmap was generated to represent the network analysis and gene expression patterns, with a focus on the cytokine and chemokine terms as identified by KEGG and GO terms. (**A**) Conducting a network analysis of cytokines and chemokines in the KEGG pathway, and (**B**) examining gene expression patterns within the KEGG category. (**C**) Analyzing the network of cytokines and chemokines using GO terms, and (**D**) representing gene expression patterns within GO terms. The data analysis was conducted using a threshold of |log2 fold change| >= 1 and FDR < 0.05.

To gain further insights into the functional characteristics of the differentially expressed genes in LPS-stimulated macrophages, we performed GO enrichment analysis, with a focus on molecular function. The analysis revealed that downregulated genes were enriched in GO terms associated with cytoskeletal activity, while upregulated genes showed enrichment in activities related to ubiquitin-protein ligases and immune-related pathways, particularly cytokine and chemokine activity (Figs. 2B, 2C, and S3). Additionally, network analysis identified genes associated with KEGG categories (Fig. 3A) and GO terms (Fig. 3C), with a specific focus on cytokines and chemokines secreted by macrophages in response to LPS stimulation. Notably, the analysis highlighted increased expression of leukemia inhibitory factors and members of the chemokine (C-C and C-X-C motifs) ligand families (Fig. 3B and 3D), suggesting that LPS stimulation can induce the secretion of diverse factors, including chemokine ligands, from macrophages.

### Validation of RNA-Seq Results by Real-Time PCR

**Fig. 4:**
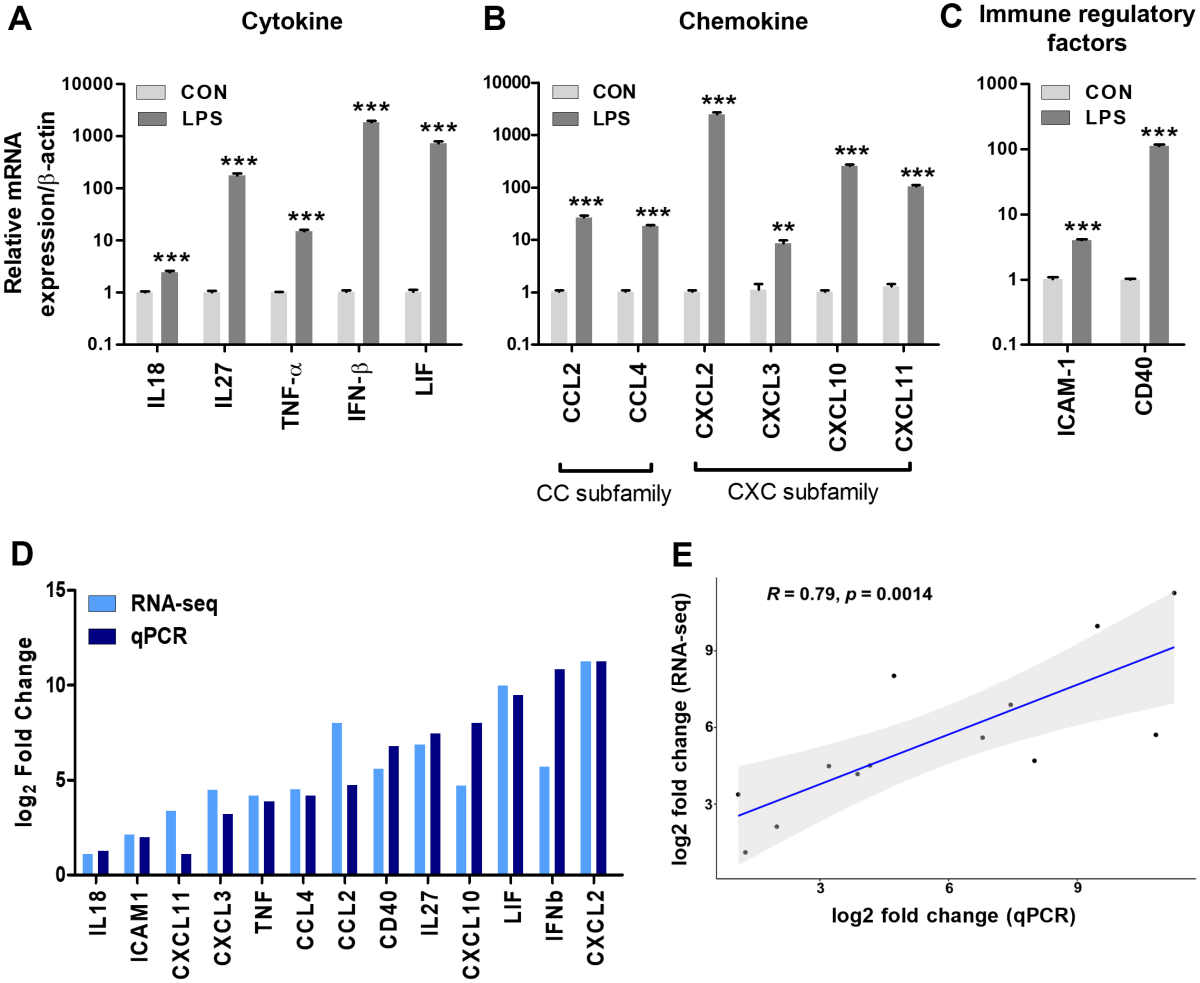
Validation of RNA-Seq results using RT-qPCR. Relative expression levels of (**A**) cytokines including IL-18, IL-27, LIF, IFN-β, and TNF-α (**B**) chemokines including CCL2, CCL4, CXCL2, CXCL3, CXCL10, and CXCL11 (**C**) immune regulatory factors including CD40 and ICAM, as determined by RT-qPCR. (**D**) Comparison of log2 fold changes obtained from RNA-Seq and RT-qPCR for selected genes. (**E**) Linear regression analysis of X-axis (log2 fold change qPCR) and Y-axis (log2 fold change RNA-seq) data showed a correlation coefficient (**R**) of 0.79. Data are presented as mean ± SEM. Statistical analysis was performed using separate tests for each gene, and the significance levels were indicated as follows: **p < 0.01, ***p < 0.001 compared with the CON group.

To validate the reliability of our transcriptomic sequencing results, we performed real-time PCR (RT-qPCR) analysis on a selection of randomly chosen secreted genes. The selected genes represented a diverse range of molecules, including cytokines (Fig. 4A; IL-18, IL-27, Leukemia inhibitory factor (LIF), Interferon (IFN)-β, and TNF-α), chemokines (Fig. 4B; CCL2, CCL4, CXCL2, CXCL3, CXCL10, and CXCL11), and immune regulatory factors (Fig. 4C; Cluster of differentiation (CD) 40 and Intercellular adhesion molecule (ICAM)-1). The RT-qPCR analysis results exhibited consistent expression patterns with those obtained from RNA-seq, as demonstrated by the log2 fold change values presented in Fig. 4D. Moreover, a correlation analysis yielded a correlation coefficient (R) of 0.79 (Fig. 4E), indicating similarity in gene expression patterns between the two methods.

### Analysis of MCM Proteome Profiles and Identification of Common Expression Patterns through RNA-Seq Analysis

**Fig. 5:**
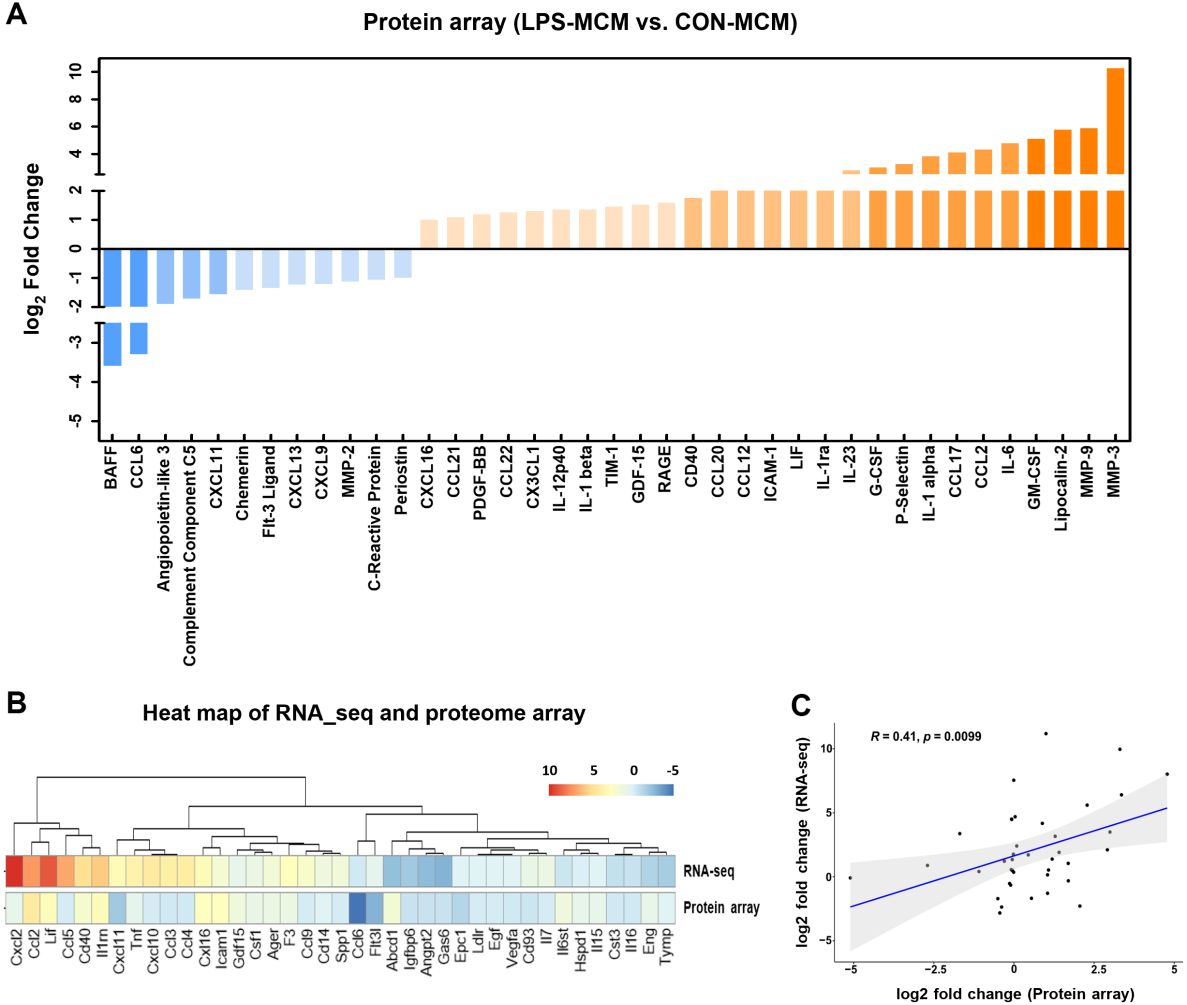
Comparative analysis of gene expression patterns and correlation between proteome cytokine and chemokine array and RNA-seq. (**A**) We examined the expression of secreted proteins through a cytokine and chemokine array, identifying proteins with |log2 fold change| >= 1. (**B**) Heatmap illustrating the expression patterns of 39 secreted factors. (**C**) Correlation analysis using the Pearson correlation method was performed to evaluate the relationships between the data sets (X-axis: log2 fold change proteome array, Y-axis: log2 fold change RNA-seq).

To assess the expression levels of secreted factors, including cytokines and chemokines, in the MCM, we conducted a proteome profile analysis (Fig. 5A). Among the 120 proteins analyzed, 45 factors exhibited upregulation, while 26 factors showed downregulation (|log2 fold change|>=1) in LPS-MCM. Notably, factors associated with muscle atrophy including IL-1α, IL-1β, growth differentiation factor (GDF)-15, and IL-6 [28-31] displayed significant increases (5.2-, 1.8-, 1.7-, and 5.6-fold change, respectively) in the LPS-MCM, suggesting their potential role in skeletal muscle atrophy. Furthermore, we examined the expression patterns of 39 secreted factors that were commonly identified in both RNA-seq analysis and proteome array experiments. This analysis allowed us to assess the correlation between their mRNA and protein expression levels (Fig. 5B). Interestingly, the correlation analysis revealed a positive correlation coefficient (R) of 0.41 (Fig. 5C) between the 39 commonly identified secretion factors in the RNA-seq analysis and proteome array experiment, further supporting the agreement between mRNA and protein expression patterns for the identified secreted factors.

## Discussion

The present study aimed to investigate the impact of macrophage-secreted factors on muscle atrophy and glucose metabolism, considering the crucial role of macrophages in tissue repair and immune regulation. Macrophages have garnered significant interest in various diseases and conditions due to their diverse functions. In this research, we employed a combination of RNA-seq and proteomic analysis to identify the pathways and factors involved in the metabolism of LPS-stimulated macrophages, providing insights into the regulation of macrophage secretion and its potential role in muscle-related diseases.

Our study focused on C2C12 myotubes exposed to LPS-MCM to simulate the effects of macrophage-derived factors on muscle cells. We observed increased expression of E3 ubiquitin ligases associated with the ubiquitin-proteasome pathway in response to LPS-MCM, indicating the activation of muscle atrophy pathways. Furthermore, LPS-MCM treatment induced an inflammatory response in the muscle cells, as evidenced by the upregulation of pro-inflammatory cytokines and mediators such as TNF-α, IL-6, iNOS, and COX-2. This inflammatory milieu, coupled with a reduction in cell diameter, suggested the potential for muscle atrophy. Notably, the amplified expression of pro-inflammatory cytokines like TNF-α and IL-6 was closely linked to a decline in muscle mass [8]. These pro-inflammatory cytokines are implicated in instigating muscle loss, partly through the activation of the nuclear factor kappa-light-chain-enhancer of activated B cells (NF-κB) transcription factor [11]. NF-κB activation has been associated with muscle loss owing to its influence on proteins like MuRF1 and inducible nitric oxide synthase, both of which are tied to genes linked with atrophy [11]. Additionally, inflammatory cytokines impact muscle protein metabolism through diverse signaling pathways, potentially impeding the Akt/mTOR signaling pathway or modulating the insulin/IGF-1 signaling cascade [11]. The convergence of this inflammatory milieu, coupled with a reduction in cell diameter, indicates a potential scenario of muscle atrophy.

Maintaining normal blood sugar levels is crucial for optimal cellular function, and disruptions in glucose uptake can have significant implications for metabolic health [15, 32]. Our study revealed that LPS-MCM significantly reduced glucose uptake in C2C12 myotubes, both in insulin-stimulated and non-insulin-stimulated conditions. These findings suggest that factors secreted by LPS-treated macrophages have the potential to interfere with glucose uptake, thus impacting glycemic control.

We performed RNA-seq analysis on LPS-treated macrophages to further understand the molecular mechanisms that may underlie this influence. The analysis revealed extensive alterations in gene expression patterns, with a substantial number of genes showing differential expression. These differentially expressed genes were not limited to immune system-related genes but also encompassed a wide array of signaling pathways associated with carbohydrate metabolism, fatty acid beta-oxidation, and other nutritional metabolic processes. This suggests that LPS stimulation not only activates the immune system in macrophages but also potentially influences nutrient metabolism pathways.

GO analysis provided further insights into the functional characteristics of the differentially expressed genes in LPS-stimulated macrophages. Downregulated genes showed enrichment in GO terms associated with cytoskeletal activity, while upregulated genes exhibited enrichment in activities related to ubiquitin-protein ligases and immune-related pathways, particularly cytokine and chemokine activity. Additionally, network analysis identified increased expression of leukemia inhibitory factors and members of the chemokine ligand families, highlighting the secretion of diverse factors from LPS-stimulated macrophages.

To validate the reliability of our transcriptomic sequencing results, we performed RT-qPCR analysis on a selection of randomly chosen secreted genes. The RT-qPCR analysis results exhibited consistent expression patterns with those obtained from RNA-seq, indicating the robustness of our findings. Moreover, correlation analysis between the commonly identified secretion factors in RNA-seq analysis and proteome array experiments revealed a positive correlation, further supporting the agreement between mRNA and protein expression patterns for the identified secreted factors.

LPS is widely known as an important factor inducing inflammatory responses [19]. However, recent evidence suggests that its impact extends beyond inflammation, encompassing various metabolic processes. Investigations focusing on LPS effects on cellular metabolism have demonstrated its involvement in the regulation of phospholipid metabolism, as evidenced by the increased incorporation of radiolabeled precursors into phosphatidylcholine observed during LPS stimulation of macrophages [33]. Additionally, studies have revealed the potential influence of LPS potential influence on lipid metabolism, as it enhances the accumulation of triglycerides and cholesterol esters in macrophages, thereby potentially contributing to foam cell formation in atherosclerosis [34]. In line with these observations, our RNA-seq results also revealed significant differences in gene expression pattern related to lipid metabolism in macrophages subjected to LPS stimulation. However, it is important to acknowledge that there may exist research findings that do not align with our RNA-seq anlysis data. Notably, recent studies have uncovered the ability of LPS ability to regulate autophagy, a cellular process involved in protein and organelle turnover, through the PI3K/Akt/mTOR pathway in macrophages [35]. However, sequencing analysis did not unveil any significant changes related to autophagy in our study. Nonetheless, these collective findings substantiate the multifaceted effects of LPS on cellular metabolism, transcending its well-established role in inflammation. While our investigation primarily focused on the secretion of cytokines and chemokines associated with these metabolic changes, it is crucial to recognize the broader significance of this issue as it can ultimately lead to the secretion caused by numerous metabolic alterations. Further explorations hold promise for gaining deeper insights into the pathophysiology of various diseases and developing targeted therapeutic strategies, as there are still numerous unexplored studies concerning the intricate interactions between LPS and metabolic pathways.

In the context of our study, we emphasize the critical importance of investigating the underlying secreted factors contributing to skeletal muscle inflammation, atrophy, and impaired glucose uptake. To comprehensively understand these factors, we conducted an array analysis of macrophage media, specifically focusing on the secreted factors following LPS stimulation. This approach provided a comprehensive overview of the presence of these factors, revealing a significant increase in their abundance. Based on our data from the proteome array, diverse cytokines were changed in MCM after LPS treatment. In particular, matrix metalloproteinase (MMP)-9, IL-6, IL-1, and GDF-15 among others were identified, and their impact on muscle health status has been extensively studied, elucidating the intricate relationship between inflammation, cytokines, and muscle health. This understanding holds significant relevance in developing therapeutic strategies to mitigate muscle wasting and insulin resistance across diverse pathological conditions. Among the upregulated factors, MMPs displayed the most significant increase. MMPs are enzymes synthesizing, degrading, and remodeling extracellular matrix (ECM) components [36]. Particularly, MMP-9 is prominently expressed in inflammatory muscle diseases and is being explored as a potential therapeutic target for such conditions [36]. Research has revealed that MMP-9 expression in response to various proinflammatory cytokines is regulated through the transcriptional activation of NF-κB and AP-1 in skeletal muscle [37]. This activation leads to ECM degradation, impairing muscle regeneration and resulting in extensive fibrosis [37]. IL-6, a pro-inflammatory cytokine, promotes muscle protein breakdown by activating the ubiquitin-proteasome system and autophagy, leading to the degradation of muscle proteins [38]. Furthermore, IL-6 inhibits the differentiation and fusion of myoblasts, impairing muscle regeneration [38]. In the context of insulin resistance, IL-6 interferes with insulin signaling pathways by activating serine kinases, such as c-Jun N-terminal kinase (JNK), which phosphorylate insulin receptor substrate proteins and disrupt insulin receptor signaling [39]. Similarly, IL-1β has been implicated in muscle atrophy and insulin resistance. IL-1β stimulates the production of other pro-inflammatory cytokines and promotes muscle protein breakdown by activating the NF-κB pathway. The upregulation of muscle-specific E3 ubiquitin ligases, such as MuRF1 and MAFbx/Atrogin-1, further contributes to muscle protein degradation [28]. In terms of insulin resistance, IL-1β interferes with insulin signaling by activating stress kinases like JNK and inhibitor of nuclear factor kappa B kinase subunit beta (IKKβ), which phosphorylate insulin receptor substrate proteins and disrupt downstream insulin signaling cascades [40]. GDF-15, a stress-responsive cytokine, has emerged as a potential player in muscle atrophy and insulin resistance. GDF-15 activates signaling pathways involved in muscle protein breakdown, such as the Bcl-2/caspase-3 pathway, contributing to muscle wasting [41]. Although the direct effects of GDF-15 on muscle insulin resistance are not fully understood, elevated GDF-15 levels have been associated with insulin resistance in various tissues, suggesting a potential indirect influence on muscle insulin sensitivity [42]. GDF-15 may induce systemic inflammation and alter energy metabolism, contributing to muscle insulin resistance. It is important to note that the effects of MMP-9, IL-6, IL-1, and GDF-15 on muscle atrophy and insulin resistance are complex and intertwined. These cytokines are part of a larger network of inflammatory mediators and signaling pathways that contribute to muscle health and metabolic regulation. Additionally, the interactions between these cytokines and other factors, such as hormonal signaling, oxidative stress, and nutritional status, further influence their effects on muscle tissue. Future research should focus on elucidating the precise molecular mechanisms by which MMP-9, IL-6, IL-1, and GDF-15 derived from macrophages exert their effects on muscle atrophy and insulin resistance. Furthermore, identifying potential therapeutic targets to modulate the activity of these cytokines may offer promising avenues for the prevention and treatment of muscle wasting and insulin resistance in various pathological conditions. It is crucial to acknowledge that numerous other factors, beyond those already identified, have shown changes that warrant further investigation. We believe that these factors may interact through diverse mechanisms, and their combined effects have the potential to generate novel impacts on skeletal muscle. Notably, the regulation of macrophage secretions holds promise for important implications in skeletal muscle health and immune balance [43, 44].

In conclusion, our study provides valuable insights into the impact of macrophage-secreted factors on skeletal muscle atrophy and glucose metabolism. Through RNA-seq, proteomic analysis, and array analysis of macrophage media, we uncovered alterations in metabolic pathways and identified factors associated with these changes. The findings contribute to the growing body of knowledge surrounding macrophage regulation and its implications for muscle-related diseases. Further investigations in this field hold the potential to provide novel insights into therapeutic targets and strategies for ameliorating muscle atrophy and metabolic disorders. Future research should aim to elucidate the specific signaling pathways and molecular mechanisms underlying the effects of cytokines and chemokines secreted by LPS-stimulated macrophages, as well as explore the dynamic interplay between macrophages and other immune cells in the context of muscle atrophy and metabolic dysregulation. Such investigations will contribute to a more comprehensive understanding of these complex processes and pave the way for targeted interventions in muscle-related diseases.

## Supplemental Materials

Supplementary data for this paper are available on-line only at http://jmb.or.kr.
