# Supplementary material for: LPS-Induced Modifications in Macrophage Transcript and Secretion Profiles Are Linked to Muscle Wasting and Glucose Intolerance
Source: J Microbiol Biotechnol. 2023 Dec 1;34(2):270–9. doi: 10.4014/jmb.2309.09037 (PMC10940789; doi:10.4014/jmb.2309.09037)
Supplement: Supplementary file 1 [file jmb-34-2-270-supple.pdf]

J. Microbiol. Biotechnol.
https://doi.org/10.4014/jmb.2309.09037

Supplementary Figures

(A)

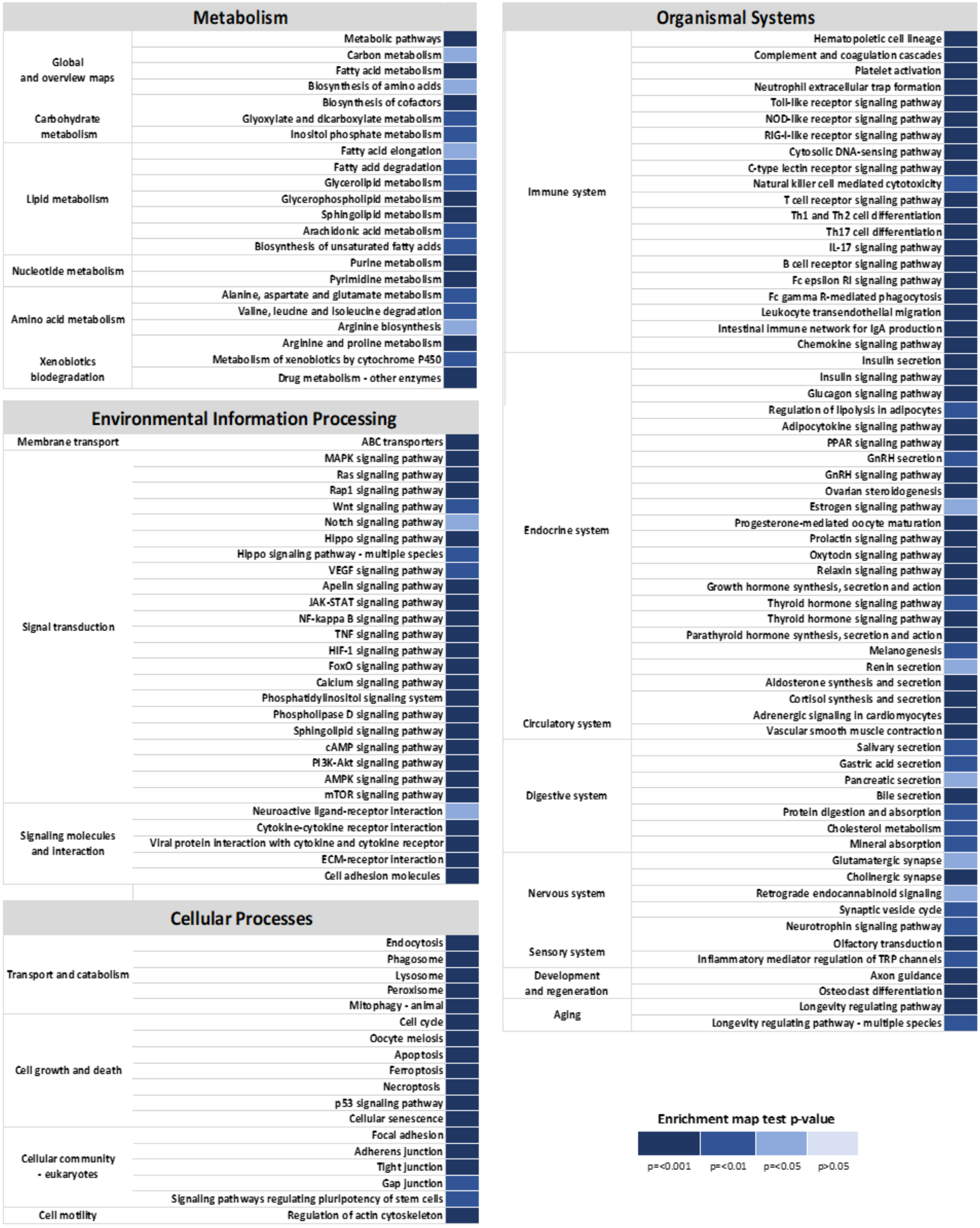

Fig. S1. LPS-stimulated macrophages undergo changes in various signaling pathways. (A) Beyond immune system-related genes, they also exhibit effects on diverse signaling pathways, including metabolism (carbohydrate, fatty acids, and other nutrient metabolism), cellular processes (cell cycle, apoptosis, etc.), and organ systems (endocrine system, nervous system, etc.).

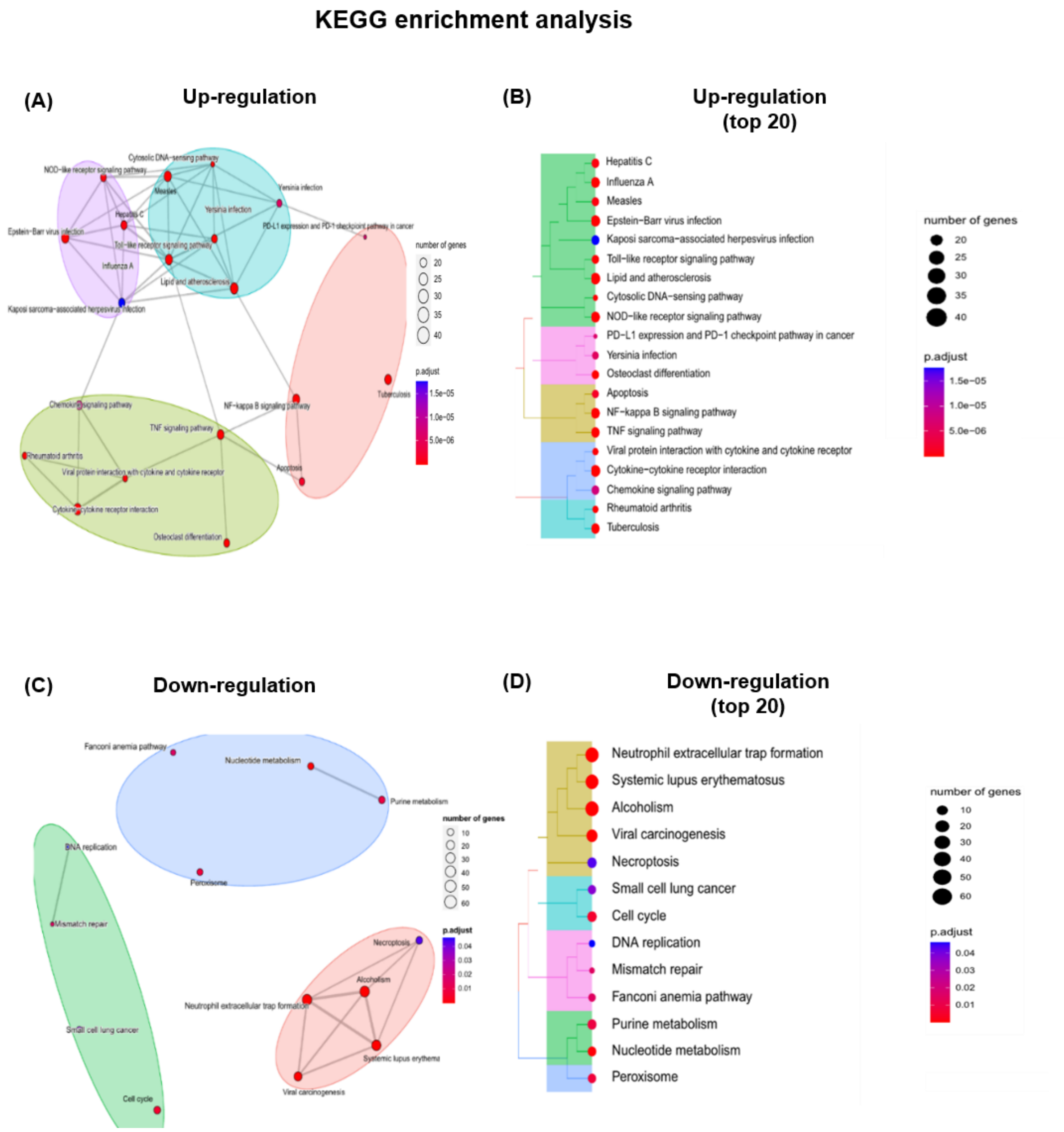

Fig. S2. KEGG enrichment analysis of differentially expressed genes in LPS-stimulated macrophages. The DEG analysis results were used to classify genes into up-regulated and down-regulated categories, and KEGG pathway analysis was conducted. **(A)** The up-regulated genes were visualized as a KEGG pathway map, and **(B)** the top 20 categories were extracted for display. **(C)** The down-regulated genes were also displayed as a KEGG pathway map, with **(D)** the 13 categories shown in a tree plot format. The data analysis was conducted using a threshold of |log2 fold change| >= 1 and FDR < 0.05.

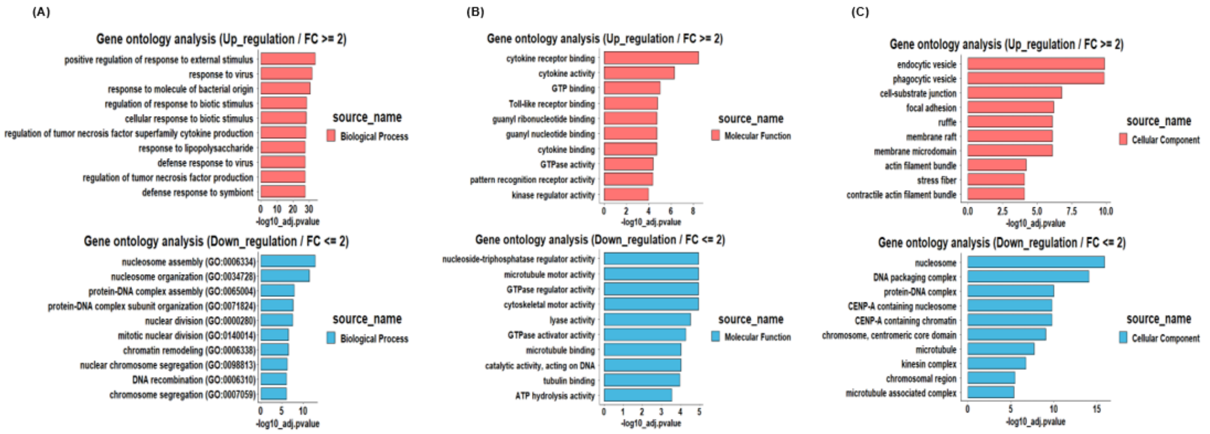

Fig. S3. GO enrichment analysis of LPS-stimulated macrophages. We performed GO enrichment analysis to investigate the functions and roles of genes in macrophages stimulated with LPS. **(A)** biological process, **(B)** molecular function, and **(C)** cellular component.
